# Synthesis of Ce-MOF/Ag composites with improved electrocatalytic activity and stability for sustainable water splitting

**DOI:** 10.1038/s41598-026-56365-0

**Published:** 2026-07-12

**Authors:** Nourhan M. Ibrahim, Ayman S. Eliwa, Mahmoud A. Hefnawy, Gehad G. Mohamed, Wafaa M. Hosny, Omar A. Fouad

**Affiliations:** 1 Chemistry Department, Faculty of Science, Cairo University, 12613 Giza, Egypt; 2 Nanoscience Department, Faculty of Basic and Applied Sciences, Egypt-Japan University of Science and Technology, New Borg El Arab, Alexandria, 21934 Egypt

**Keywords:** Ce-MOF, Silver nanoparticles, Nano composites, Water splitting, Hydrogen production, Chemistry, Environmental sciences, Materials science

## Abstract

The increasing global demand for clean and sustainable energy has intensified the need for efficient hydrogen production via water splitting, yet the development of cost-effective and high-performance electrocatalysts remains a major challenge. In this context, the present study focuses on the design of an advanced MOF-based composite to enhance catalytic efficiency and stability. This study was focused on preparing an MOF-based composite to enhance its efficiency in water splitting applications. The MOF framework was enhanced with additional active ingredients to boost its overall performance, stability, and catalytic activity. Developing such advanced composites is crucial for addressing the challenges of energy conversion and hydrogen generation via water splitting, which is considered a clean and sustainable route for future energy systems. A metal–organic framework (MOF) containing cerium and MOF-Ag composites have been synthesized using the green methodology. The generated materials were examined using several analytical methods, including contact angle, powder X-ray diffraction (PXRD), FTIR, Brunauer-Emmett-Teller (BET), transmission electron microscope (TEM) and scanning electron microscopy (SEM). The average particle size of the synthesized MOF-Ag composites ranges from 36.7 to 41.3 nm, which is confirmed by the SEM data in combination with the Gaussian mixture model. The BET and contact angle data confirm the mesoporous and hydrophilic characteristics of the MOF-Ag composites. The activity of the modified Ce-MOF and MOF-Ag composites electrode was utilized for water-splitting purposes. The current flowing through the electrodes lasts for five hours. The electrodes (MOF, MOF-Ag1, MOF-Ag2, and MOF-Ag3) attained the current density of 10 mA cm^− 2^ at potentials of ‒1.16, ‒0.88, ‒0.9, and ‒0.94 V (vs. RHE) for HER and at potentials of 2.5, 2.07, 2.12, and 2.18 V (vs. RHE) for OER, respectively. Additionally, each of the changed surfaces (MOF, MOF-Ag1, MOF-Ag2, and MOF-Ag3) had its Tafel slopes calculated¸ yielding values of 150, 76, 86, and 91 mV dec^− 1^ for HER and 178, 90, 128, and 138 mV dec^− 1^ for OER.

**Supplementary Information:**

The online version contains supplementary material available at 10.1038/s41598-026-56365-0.

## Introduction

Modern civilization is based on sufficient supply of energy for socio-economic progress where the primary energy source has been fossil fuels, yet these are steadily disappearing from the record. Because using fossil fuels releases greenhouse gases and pollutants into the atmosphere, it contributes to global climate change, so that it is crucial to promote the utilization of sources of sustainable energy to satisfy both the environmental factor and the world’s steadily rising energy demand^1^. Global clean energy demands can be effectively addressed using the promising approach of hydrogen energy generation combined with less energy consumption^2,3^. One of the biggest and most significant concerns in the modern world is the search for alternative energy sources^4–8^. The hydrogen evolution reaction (HER) is an electrochemical process in which hydrogen gas is generated from water through the application of electricity. As a sustainable and carrier of renewable energy, hydrogen can be employed in multiple fields, including fuel cell technology, electricity generation, and industrial synthesis^9,10^. Adoption of HER provides a viable approach to reducing dependency on fossil fuels, which are the main source of emissions of greenhouse gases and the degradation of air quality^11^. Because water is used as the starting material, this process avoids the environmental and health hazards linked to fossil fuel extraction and transportation. Furthermore, HER provides the effective incorporation of renewable energy sources, for instance, integrating solar and wind energy into the energy grid^12^. Electrocatalysis has emerged as a key approach with broad applications, including in sensors, photovoltaic devices, water-splitting technologies and fuel cells^13–15^. Among these, electrocatalytic hydrogen evolution (EHE) represents an encouraging path to produce pure hydrogen fuel, accomplished by using an applied electric current to break down water into hydrogen and oxygen. Despite its potential, the process demands highly active and durable catalysts capable of driving the reaction efficiently at minimal overpotentials while sustaining elevated current densities^16^.

Recently, environmental problems, such as global warming, have become more severe; thus, there is a requirement to implement sustainable development goals in materials processing^17^. Extensive attention has been paid to the nanostructured materials owing to their wide range of important and significant applications and interesting and unique properties^18,19^.

Within this framework, metal–organic frameworks (MOFs) have attracted a lot of attention. as electrochemically active catalysts and have been widely applied in diverse electrochemical systems, including fuel cells, lithium batteries^20–22^, supercapacitors^23–25^, and water-splitting technologies^26,27^. Their effectiveness arises from distinctive structural and electrochemical features, like crystallinity, exceptionally large pore volume, high surface area, high aspect ratio, tunable linkers or metal nodes, presence of redox-active metal centers, excellent stability, abundance of active sites, notable electrocatalytic activity, and well-defined morphology. Moreover, the accessibility of molecular adsorption sites in MOFs facilitates host–guest interactions, enabling efficient material capture through both chemisorption and physisorption processes^28,29^. Currently, materials science stands out as an exciting interdisciplinary discipline that explores the design and application of different materials across a wide range of fields. Although the concept is not entirely new, a breakthrough was achieved in 1995 when Yaghi and colleagues revealed the preparation of the first MOF, a special class of porous materials constructed from inorganic metal centers coordinated with organic linkers^30^. The significance of MOFs continues to grow annually, largely owing to their ease of modification, it has led to their becoming one of the most researched material classes. MOFs exhibit remarkable chemical and physical characteristics suitable for diverse applications, and these properties can be further enhanced through different approaches. Strategies include the introduction of functional groups^31^, variation of organic linkers^32^, impregnation with active components^33^, post-synthetic ligand or ion exchange^34^, and the development of composites with compatible materials^35–39^. Composites are multi-phase systems composed of two or more distinct constituents, with at least one continuous phase^40^. Recently, MOF-based composites have attracted a lot of interest due to their many uses, particularly in adsorption. Although relatively new, this field has seen a growing number of studies demonstrating successful synthesis and promising functionalities. Combining MOFs with other materials can significantly influence synthesis kinetics^41^, morphology^34,42^, stability^43–45^, physicochemical behavior, and overall performance in various applications^46,47^. Sahar Zinatloo et al. (2019) reported the green synthesis of a recyclable magnetic nanocomposite, CoFe₂O₄@SiO₂@Dy₂Ce₂O₇, using environmentally friendly preparation methods in which grape juice was employed as a natural fuel^48^. Sahar Zinatloo et al. (2020) reported the green synthesis of Dy₂Sn₂O₇ nanoparticles using *Ficus carica* extract as a natural fuel through an environmentally friendly route. The prepared nanostructures showed controlled morphology, high purity, and suitable surface properties^49^. Cerium(IV)-based MOFs have attracted growing attention considering their vast surface area, flexible structural composition, and reasonable chemical and thermal stability^50,51^. Additionally, besides their academic relevance, several industrial considerations motivated this study: (i) cerium is the most prevalent rare-earth element, making it a cost-effective option for large-scale MOF production; (ii) Ce(IV)-MOFs show potential in areas like heterogeneous redox catalysis, photocatalysis and luminescence, the latter connected to the availability of unoccupied 4f orbitals that lie low; and (iii) not enough research has been done on the production of Ce(IV)-MOFs at room temperature^52,53^, offering opportunities for enhancing sustainability. Although Ce-based MOFs are considered stronger candidates for industrial deployment compared with some other analogues^50,54^, their practical commercialization requires synthesis routes that are economically viable, environmentally benign, and energy-efficient objectives that are still challenging to fully realize. As one of the remarkable oxide materials based on rare earth elements, ceria (CeO_2_) is known for its unique structural, optical, and redox features and thermal stability^55^. Lammert et al.^51^ reported the successful synthesis of an isoreticular series of porous cerium-based MOFs with the UiO-66-type framework. The study identified suitable conditions for the self-assembly of the [Ce₆O₄(OH)₄]¹²⁺ cluster, enabling the preparation of Ce-MOFs with different linker lengths and functionalized organic ligands. XANES analysis confirmed the presence of Ce⁴⁺ ions, while Ce-UiO-66-BDC exhibited the highest chemical and thermal stability. In addition, preliminary catalytic investigations demonstrated its effectiveness as a co-catalyst with TEMPO in alcohol oxidation reactions, highlighting the promising catalytic potential of cerium-based MOFs^56^.

Despite the rapid development of MOF-based electrocatalysts for water splitting, key challenges remain, including poor intrinsic electrical conductivity, limited exposure of active sites, and insufficient long-term stability under operating conditions. In addition, the use of environmentally benign synthesis routes for constructing high-performance MOF-based composites is still relatively underexplored. In this work, we address these limitations through the rational design of a cerium-based MOF integrated with silver nanoparticles via a green synthesis approach. The novelty of this study lies in the dual strategy of enhancing both the physicochemical and electrochemical properties of the MOF system: (i) by incorporating conductive Ag nanoparticles to improve charge transfer kinetics and catalytic activity, and (ii) by tailoring surface characteristics such as porosity and hydrophilicity to facilitate electrolyte accessibility and interfacial reactions. Furthermore, this work provides a systematic correlation between structural features (particle size distribution, mesoporosity, and surface wettability) and electrocatalytic performance toward both HER and OER. The resulting MOF–Ag composites not only demonstrate improved activity and stability compared to the pristine MOF, but also establish a scalable and sustainable pathway for designing efficient, non-noble metal-based electrocatalysts for hydrogen generation.

## Experimental

### Plant extract preparation

Dried clove buds (10.0 g) were first rinsed with distilled water to remove surface impurities and then added to 100 mL of distilled water. The mixture was heated to boiling and maintained under gentle boiling for 60 min. After cooling naturally to room temperature (~ 25 °C), the resulting extract was filtered through Whatman No. 1 filter paper to remove solid residues. The clear filtrate was collected and stored at 4 °C for subsequent use as a reducing agent, and it was utilized within 24 h of preparation^51,57,58^.

### Green silver nanoparticle preparation

Silver nanoparticles were synthesized via a green reduction method using the prepared clove extract. Briefly, 50 mL of clove extract was placed under magnetic stirring at room temperature, and 10 mL of 1.0 M aqueous AgNO₃ solution was added dropwise. The reaction mixture was continuously stirred for an additional 30 min following complete addition. The formation of a grey precipitate indicated the generation of AgNPs. The suspension was allowed to settle, and the supernatant was decanted. The obtained precipitate was washed three times with distilled water (20 mL each) to remove residual impurities and then dried under vacuum at 60 °C for 12 h.

### Preparation of linker solution

Terephthalic acid (H₂BDC, 0.25 g) was dispersed in 10 mL of distilled water and stirred at room temperature for 30 min. Subsequently, a 0.1 M NaOH solution was added dropwise until complete dissolution of the ligand was achieved, resulting in a clear solution with a pH in the range of 7–8. The solution was further stirred to ensure homogeneity before use in MOF synthesis.

### Preparation of Ce(IV)-MOF

Cerium(IV) ammonium nitrate ((NH₄)₂Ce(NO₃)₆, 1.0 g) was initially dissolved in 3.5 mL of acetic acid, followed by the addition of 10 mL of distilled water and 24 mL of ethanol under continuous stirring to obtain a homogeneous solution. The resulting cerium precursor solution was then added dropwise to the previously prepared linker solution under constant stirring at 600 rpm. The reaction mixture was maintained at room temperature for 4 h to allow the formation of the Ce(IV)-MOF. The resulting solid product was collected by centrifugation at 6000 rpm for 10 min and subsequently washed sequentially with DMF (1 × 20 mL), distilled water (3 × 20 mL), and ethanol (3 × 20 mL). The washing process was repeated with ethanol to ensure removal of unreacted species, followed by a final wash with acetone (20 mL). The purified material was dried under vacuum at 60 °C overnight^51,59^.

### Preparation of MOF-Ag composites

MOF-Ag composites were prepared by physically mixing the synthesized Ce(IV)-MOF with AgNPs at different weight ratios while maintaining a constant amount of MOF. Specifically, three composites were prepared with MOF: AgNPs ratios of 1:1, 1:0.8, and 1:0.6, denoted as MOF-Ag1, MOF–Ag2, and MOF-Ag3, respectively. Each mixture was dispersed in 20 mL of ethanol and subjected to ultrasonic treatment (40 kHz, 150 W) for 30 min to ensure uniform distribution and effective integration of the components. The resulting suspensions were then evaporated at 60 °C until complete removal of the solvent, yielding the final composite materials.

### Preparation of electrodes

For electrode fabrication, 10 mg of each synthesized material (Ce(IV)-MOF, MOF–Ag1, MOF–Ag2, and MOF–Ag3) was dispersed in 1 mL of ethanol and sonicated for 15 min to obtain homogeneous suspensions. Prior to modification, the glassy carbon electrode (GCE) was polished with alumina slurry, rinsed thoroughly with distilled water, and dried under ambient conditions. Subsequently, 20 µL of each suspension was drop-cast onto the surface of the GCE and allowed to dry at room temperature. Electrochemical measurements were performed in a 1.0 M acidic solution.

## Results and discussion

### Characterization

The MOF-Ag composites have been generated, dried, and subjected to various characterizations utilizing BET, SEM, XRD and contact angle, as mentioned in the experimental section.

#### PXRD analysis

**Fig. 1:**
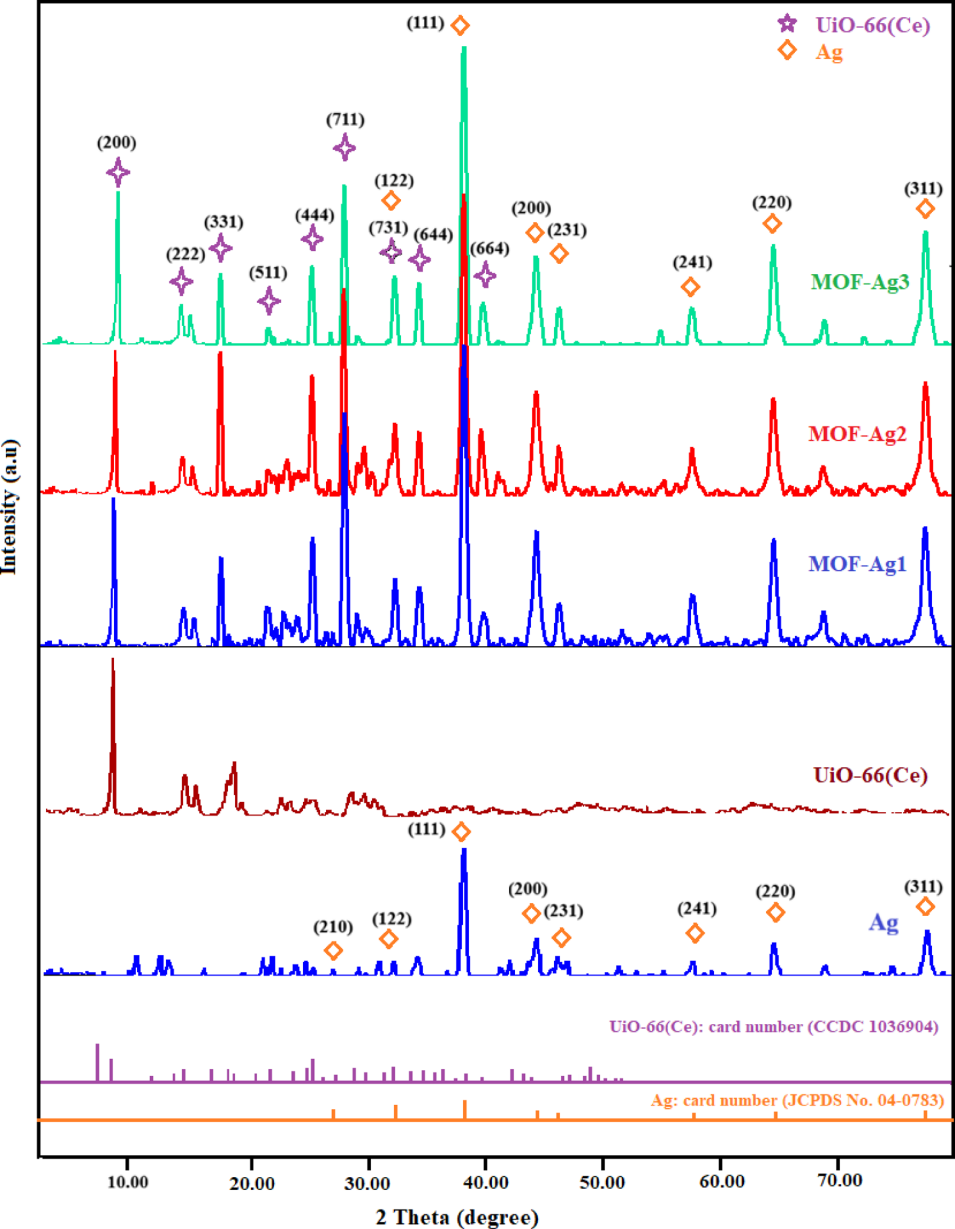
XRD chart for MOF, green silver and MOF-Ag composites (MOF-Ag1, MOF-Ag2 and MOF-Ag3).

The X-ray diffraction (XRD) patterns of the synthesized compounds were analyzed to investigate their crystalline structure and phase purity. Figure 1. displays the PXRD patterns of green Ag, Ce-MOF and the MOF-Ag composites (MOF-Ag1, MOF-Ag2, and MOF-Ag3). The presence of sharp and high-intensity diffraction peaks indicates the high crystallinity of the synthesized materials. The PXRD patterns of the MOF-Ag composites were compared with those of pristine Ce-MOF and Ag nanoparticles to confirm the successful formation and purity of the composites.

The characteristic diffraction peaks of Ce-MOF appeared at 2θ values of 8.5°, 14.38°, 17.9°, 21.49°, 28.78°, 29.69°, 31.96°, 34.41°, and 39.34°, corresponding to the crystallographic planes (200), (222), (331), (511), (444), (711), (731), (644), and (664), respectively. These peaks are consistent with the cubic crystal structure belonging to the Fm-3 m space group with a lattice parameter of a = 21.47 Å and agree well with CCDC card No. 1,036,904^51^. In addition, the diffraction peaks observed at 27.81°, 32.16°, 38.12°, 44.31°, 46.21°, 57,39°, 64.4°, and 77.5° correspond to the (210), (122), (111), (200), (231), (241), (220), and (311) planes of face-centered cubic metallic Ag nanoparticles, respectively, in agreement with JCPDS file No. 04–0783^60,61^.

The coexistence of diffraction peaks corresponding to both Ce-MOF and metallic Ag confirms the successful incorporation of Ag nanoparticles into the MOF framework without significant structural distortion. Furthermore, the absence of additional impurity peaks indicates the high phase purity of the synthesized composites. The clear and sharp diffraction peaks further demonstrate the excellent crystallinity of the prepared powders. Jacobsen et al. (2019) reported that Ce-MOF composites exhibit sharp and intense diffraction peaks, indicating high crystallinity and phase purity of the synthesized structures^62^. Similarly, Zhang et al.^63^ confirmed the incorporation of Ag nanoparticles into MOF frameworks through the appearance of characteristic diffraction peaks corresponding to both the parent MOF and metallic Ag phases^63^.

The crystallite dimensions of the synthesized green Ag, Ce-MOF and MOF-Ag composites were determined utilizing the Debye–Scherrer equation, relying on the full width at half maximum (FWHM) of the most prominent diffraction peaks (Table 1). The results indicated that the crystallite sizes of green Ag, Ce-MOF, MOF-Ag1, MOF-Ag2, and MOF-Ag3 varied from 15.61 to 19.04 nm, 11.38 to 33.52 nm, 18.12 to 34.16 nm, 13.12 to 26.68 nm, and 8.29 to 27.29 nm, respectively, with average crystallite sizes of 18.75, 22.14, 21.81, 21.02, and 19.01 nm. The measured nanoscale crystallite dimensions validate the effective synthesis of nanocrystalline Ag, Ce-MOF and MOF-Ag composites. Furthermore, the comparatively diminutive crystallite sizes may enhance electrocatalytic performance by enhancing the surface-active sites and enabling charge transfer during water-splitting reaction.

**Table 1:** The crystallite size of MOF, green silver and MOF-Ag composites (MOF-Ag1, MOF-Ag2 and MOF-Ag3).

Nano composite	Minimum crystallite size (nm)	Maximum crystallite size (nm)	Average crystallite size (nm)
Ag	15.61	19.04	18.75
Ce-MOF	11.38	33.52	22.14
MOF-Ag1	18.12	34.16	21.81
MOF-Ag2	13.12	26.68	21.02
MOF-Ag3	8.29	27.29	19.01

#### FTIR

**Fig. 2:**
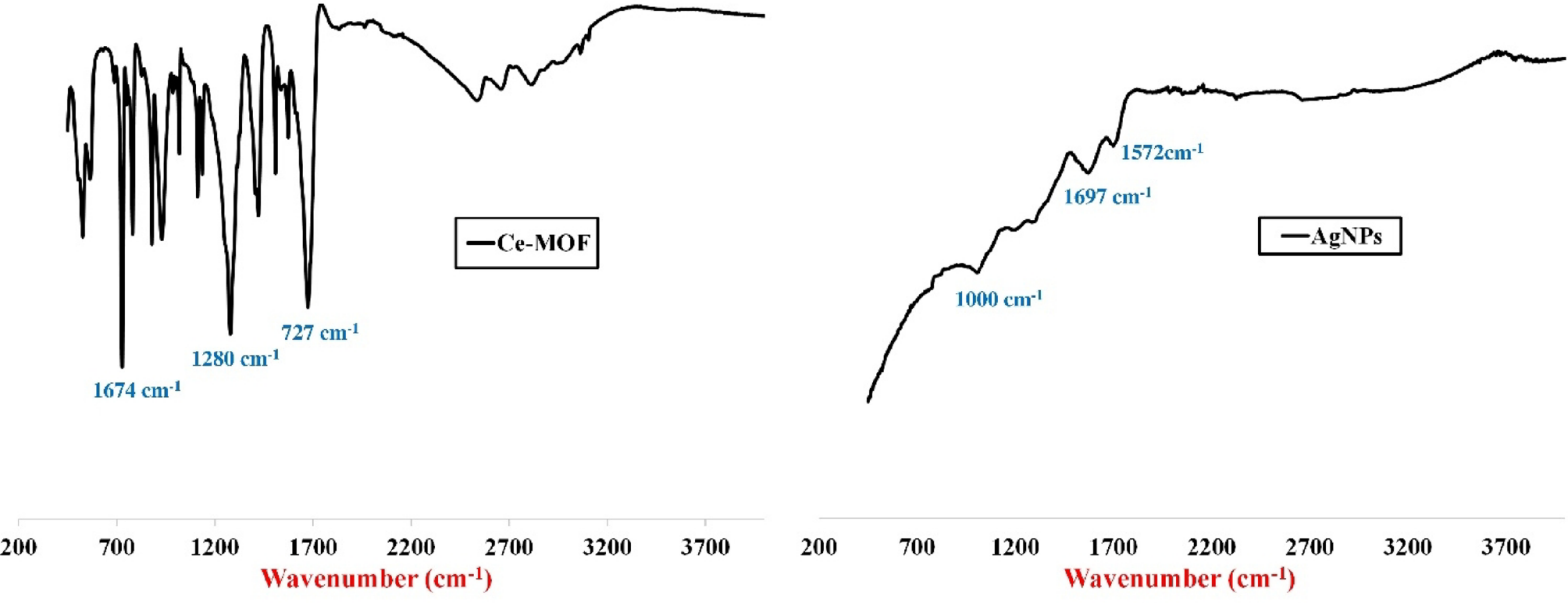
FTIR chart for Ce-MOF and green silver.

The FTIR spectra of the synthesized Ce-MOF and AgNPs are presented in Fig. 2. For the Ce-MOF spectrum, the characteristic absorption band observed at 1674 cm⁻¹ can be attributed to the stretching vibration of the carbonyl group (C = O) of the organic linker coordinated with the cerium ions. The band appearing at 1280 cm⁻¹ is assigned to the C–O stretching vibration, confirming the presence of oxygen-containing functional groups within the MOF framework. In addition, the absorption peak at 727 cm⁻¹ corresponds to the metal–oxygen (Ce–O) vibration, indicating the successful formation of the cerium-based metal–organic framework structure^64^.

For the AgNPs spectrum, the broad absorption observed around 1000 cm⁻¹ is attributed to C–O or C–N stretching vibrations originating from biomolecules or phytochemicals acting as reducing and stabilizing agents during the green synthesis process. The peaks located at 1697 cm⁻¹ and 1572 cm⁻¹ are associated with carbonyl (C = O) and aromatic C = C stretching vibrations, respectively. These functional groups confirm the interaction of organic compounds from the plant extract with the surface of silver nanoparticles, contributing to nanoparticle stabilization and preventing aggregation^65^.

Overall, the FTIR results confirm the successful synthesis of both Ce-MOF and AgNPs and reveal the presence of functional groups responsible for structural stability and enhanced electrochemical behavior.

#### SEM images

**Fig. 3:**
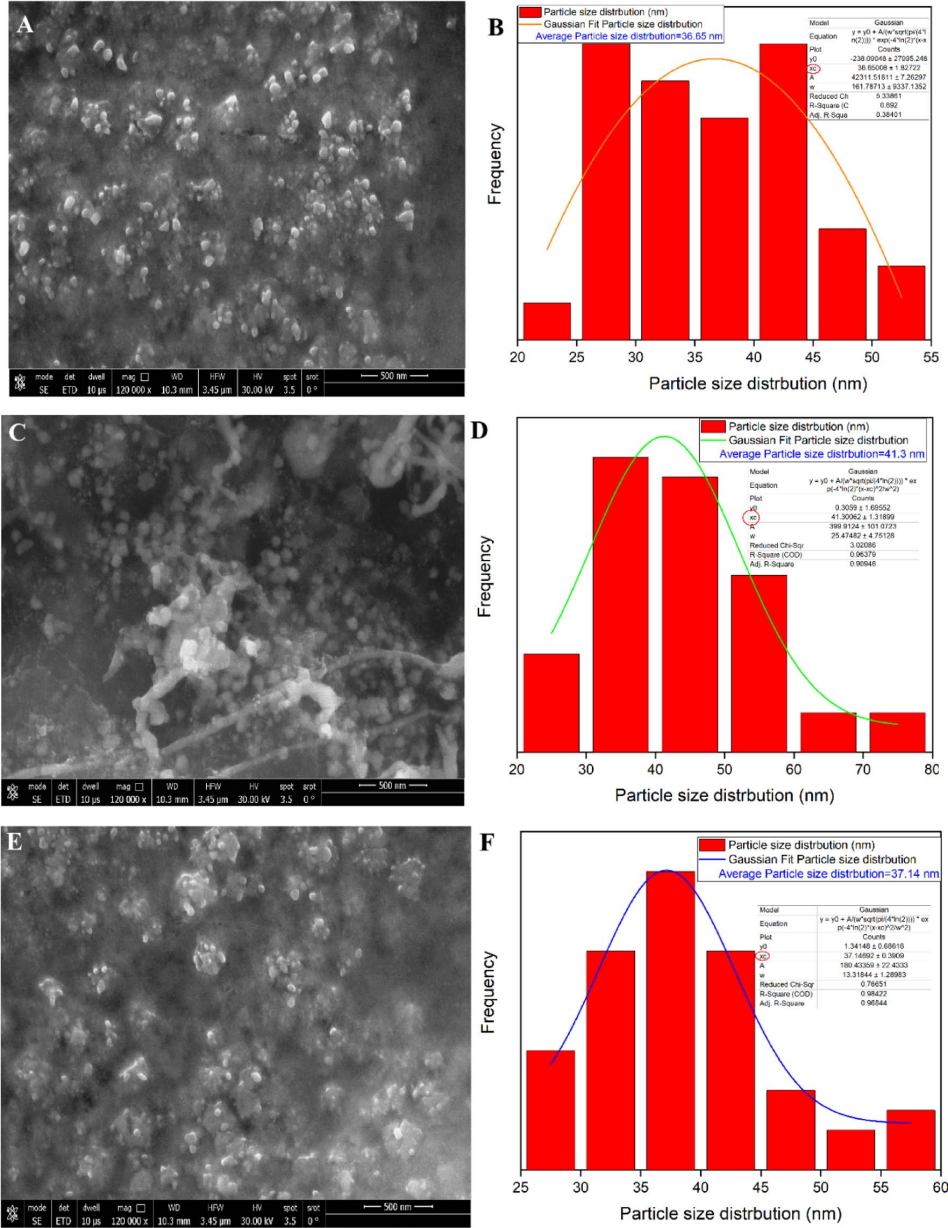
SEM images of synthesized MOF-Ag composites (**A**) MOF-Ag1, (**C**) MOF-Ag2 and (**E**) MOF-Ag3 and their particle size distributions (**B**) MOF-Ag1, (**D**) MOF-Ag2 and (**F**) MOF-Ag3.

The SEM images of the produced MOF-Ag composites (MOF-Ag1, MOF-Ag2, and MOF-Ag3), shown in Fig. 3 (A, C, and E), showed the crystals are very small, in the nanometer range. As the scale bar shows, the crystals appear semi-spherical. In some areas, they appear as agglomerates or clusters of nanoparticles. In addition, the surface appears rough and covered with protruding particles distributed in a non-uniform manner. The synthesized MOF-Ag composites were shown to contain a range of particles and pore diameters in scanning electron microphotographs (SEM). To assess the distribution of particle sizes, Java 1.8.0 172 and ImageJ (1.53e) were used to generate a Gaussian mixture model and histogram. The findings are shown in Fig. 3 (B, D, and F). proving that MOF-Ag composites (MOF-Ag1, MOF-Ag2 and MOF-Ag3) exhibited average particle sizes of 36.7, 41.3, and 37.1 nm, respectively, confirming that the synthesized MOF-Ag composites possessed a well-defined crystalline structure. Li et al. reported that Ag@MOF-801 composites revealed spherical Ag nanoparticles adorning the facets of octahedral MOF-801 structures, confirming successful Ag deposition at the nanoscale^66^. Nikmehr et al. investigated Zn-MOF morphology and implemented histogram and Gaussian fitting to SEM-derived particle size data to extract mean particle dimensions^67^. Collectively, these studies validate both our morphological observations and quantitative approach especially the combined use of SEM, ImageJ, and Gaussian fitting to elucidate the nanoscale particle size distributions of MOF-Ag composites.

#### TEM images

**Fig. 4:**
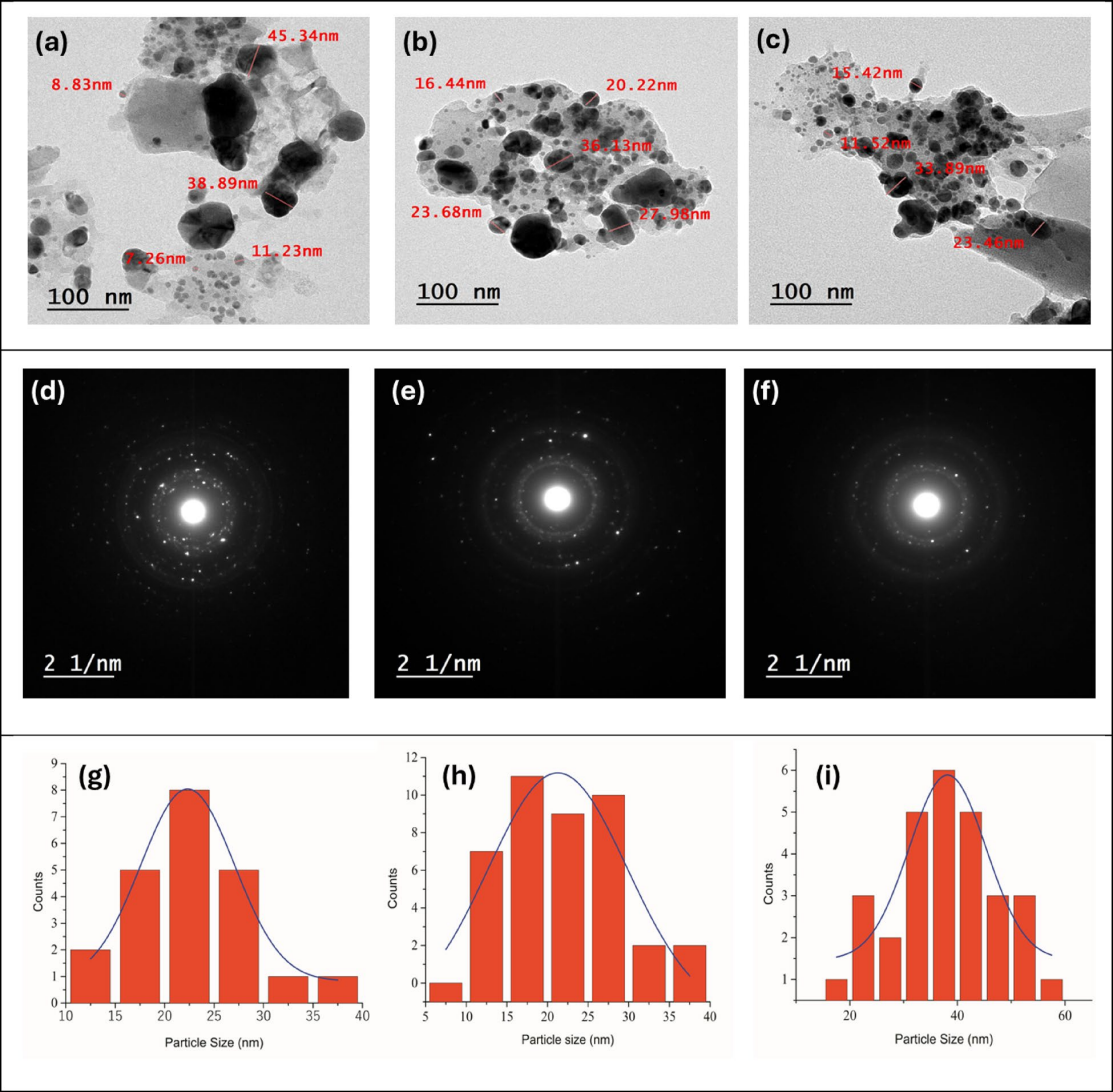
TEM micrographs of the prepared Ag-incorporated MOF composites: (**a**) MOF–Ag1, (**b**) MOF–Ag2, and (**c**) MOF–Ag3; corresponding SAED patterns of (**d**) MOF–Ag1, (**e**) MOF–Ag2, and (**f**) MOF–Ag3; and particle-size distribution histograms of (**g**) MOF–Ag1, (**h**) MOF–Ag2, and (**i**) MOF–Ag3.

The HR-TEM images of MOF‒Ag1, MOF‒Ag2, and MOF‒Ag3 Fig. 4 confirm the successful incorporation and dispersion of Ag nanoparticles within the MOF matrix. The particle size distributions were analyzed using ImageJ software (version 1.53e) based on TEM micrographs recorded at different scales^68,69^.

The TEM images of MOF–Ag1, MOF–Ag2, and MOF–Ag3 show the successful formation and distribution of Ag nanoparticles on/within the MOF matrix. The Ag particles appear as dark spherical or quasi-spherical nanodomains due to their higher electron density compared with the MOF support.

For MOF–Ag1 Fig. 4a, the particles are relatively dispersed but show some degree of aggregation. The measured particle sizes vary widely, approximately from **5** to 39 nm, indicating a broad particle-size distribution. This is also supported by the histogram Fig. 4g, where most particles are concentrated around 20–25 nm, but larger particles are present. The broad distribution may be attributed to partial nucleation and growth of Ag nanoparticles at different sites on the MOF surface.

For MOF–Ag2 Fig. 4b, Ag nanoparticles are more uniformly distributed, with most measured particles falling within the range of approximately 16–28 nm. The particle-size distribution Fig. 4h shows a relatively regular Gaussian-like profile, suggesting improved dispersion and more controlled nanoparticle growth compared with MOF–Ag1. The higher density of dark particles indicates increased Ag incorporation while still maintaining nanoscale particle formation.

For MOF–Ag3 Fig. 4c, the Ag nanoparticles remain well distributed, although localized clustering can be observed in some regions. The measured particle sizes are mostly in the range of about 15–25 nm, and the histogram Fig. 4i confirms that the majority of particles are centered around the mid-nanometer range. This suggests that increasing Ag content promotes the formation of more Ag nanodomains, while the MOF framework helps restrict excessive particle growth.

The SAED patterns of MOF–Ag1, MOF–Ag2, and MOF–Ag3 Fig. 4(d–f) display concentric diffraction rings with bright spots, confirming the polycrystalline nature of the Ag-containing MOF samples. The presence of ring patterns indicates that the Ag nanoparticles are crystalline and randomly oriented within the MOF matrix. The coexistence of diffuse and sharp diffraction features may be related to the contribution of the MOF framework together with crystalline Ag nanodomains.

Furthermore, the selected area electron diffraction (SAED) patterns corresponding to MOF‒Ag1, MOF‒Ag2, and MOF‒Ag3 Fig. 4 displayed distinct bright concentric rings accompanied by diffraction spots, confirming the crystalline nature and high crystallinity of the synthesized Ag-loaded MOF nanocomposites^70,71^. The diffraction features further verify the successful formation of nanoscale crystalline domains within the Ce-MOF framework. Overall, the TEM and SAED analyses collectively confirm the successful synthesis of nanoscale Ag/Ce-MOF composites with well-dispersed crystalline Ag nanoparticles incorporated into the MOF architecture.

#### BET analysis

**Fig. 5:**
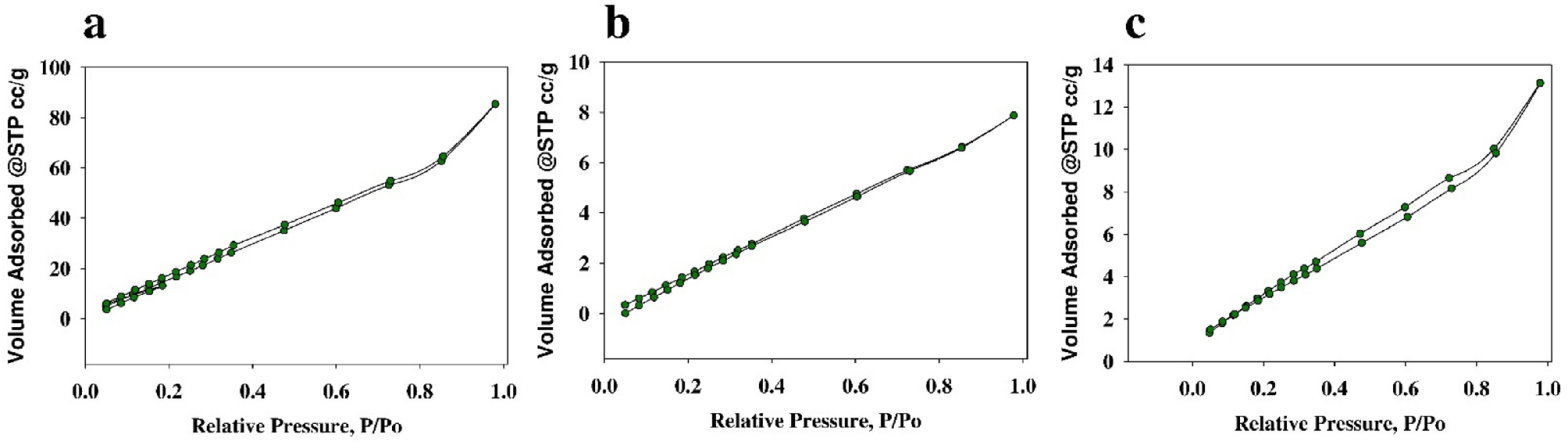
Adsorption–desorption isotherm for synthesized MOF-Ag composites (**a**) MOF-Ag1, (**b**) MOF-Ag2 and (**c**) MOF-Ag3.

A image of the BET was displayed in Fig. 5. N_2_ adsorption was utilized to quantify the porosity and volumetric surface area of the produced MOF-Ag composites. As shown in Fig. 5, standard N_2_ adsorption-desorption experiments were carried out at 77 K to investigate the pore volume, pore structure and surface area of MOF-Ag composites (MOF-Ag1, MOF-Ag2, and MOF-Ag3). After analysis, the surface areas of the BET were discovered to be 104.02, 16.29, and 16.13 m² g⁻¹, with average pore sizes of 2.54, 1.50, and 2.52 nm, and total pore volumes computed as 0.13, 0.01, and 0.02 cm³ g⁻¹ for samples MOF-Ag1, MOF-Ag2 and MOF-Ag3, respectively. According to this data, the produced nano composites (MOF-Ag1 and MOF-Ag3) are mesoporous. However, the microporous nature of (MOF-Ag2) was confirmed with a large surface area, suggesting a notable enhancement in catalytic performance because of the abundance of active sites^72,73^.

#### Contact angle

**Fig. 6:**
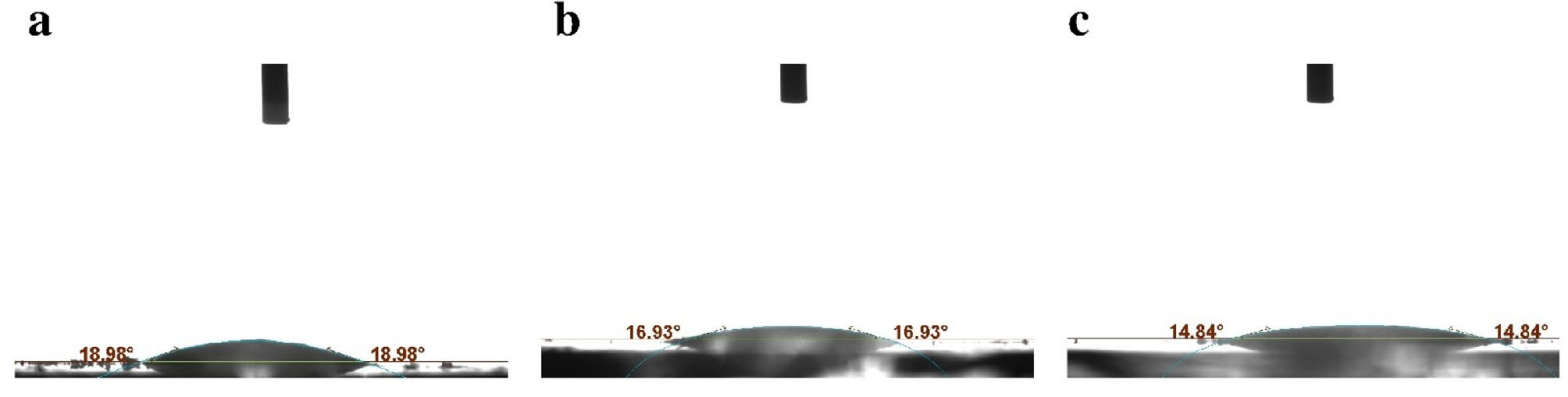
Contact angle of MOF and MOF-Ag composites (**a**) MOF-Ag1, (**b**) MOF-Ag2 and (**c**) MOF-Ag3.

Utilizing the CA computation, the lipophilicity of the recently synthesized MOF-Ag composites (MOF-Ag1, MOF-Ag2 and MOF-Ag3) has been assessed Fig. 6. and the average CA was found to be 18.98°, 16.93°, and 14.84° for MOF-Ag1, MOF-Ag2 and MOF-Ag3, respectively. Considering that these values were much lower than 90°, the hydrophilicity of the produced materials suggested a very strong affinity for water^74^. As the amount of AgNPs decrease the angle decrease, so the best one that show hydrophilicity is MOF-Ag3 due to it has the smallest CA.

### Water splitting applications

#### Cyclic voltammetry (CV) and surface acitivation

**Fig. 7:**
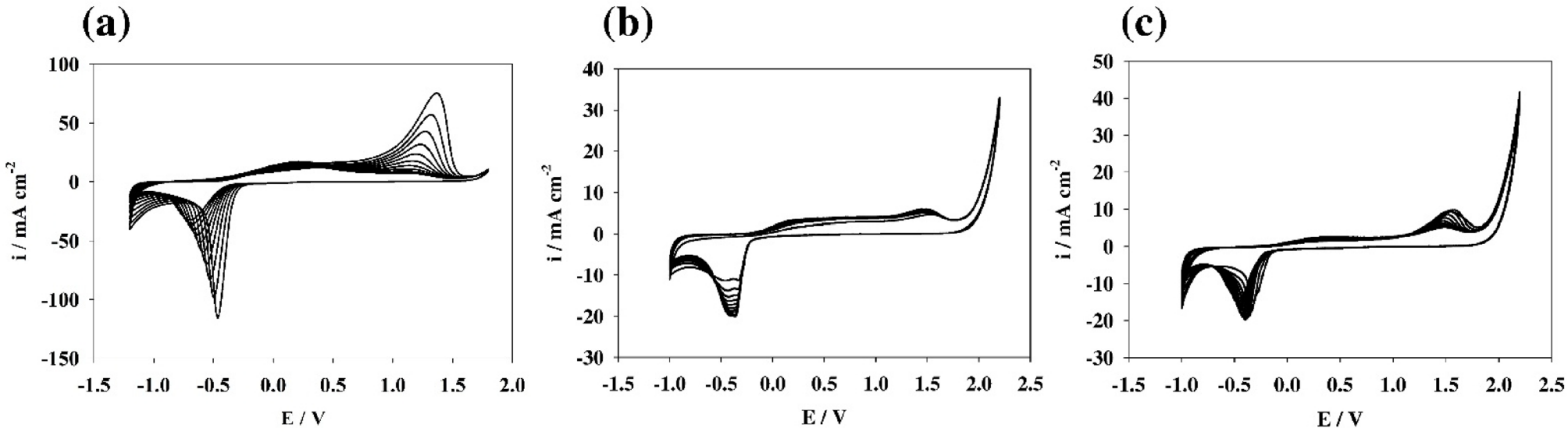
CV of modified electrodes (**a**) MOF-Ag1, (**b**) MOF-Ag2 and (**c**) MOF-Ag3 in 1 M H_2_SO_4_.

The electrochemical activity of the modified MOF-Ag electrodes (MOF-Ag1, MOF-Ag2, and MOF-Ag3) was evaluated by CV in a 1 M H_2_SO_4_ solution.The modified electrodes were first triggered in the solution to produce the electrochemically active species. Therefore, in the acidic medium, the activation step was conducted within the potential range of − 0.8 to 2.4 V (vs. RHE). Figure 7 shows 10 cycles performed on modified GC/MOF-Ag electrodes in 1 M H_2_SO_4_ solution at a scan rate of 100 mV s^− 1^. Regarding the three electrodes, two oxidation peaks were detected: a sharp one at 1.75 V (vs. RHE) and a weak one at 0.70, 0.45, and 0.42 V, respectively. However, at potentials of ‒0.05 and − 0.10 V (vs. RHE), a single reduction peak was observed, respectively, for MOF-Ag1, MOF-Ag2, and MOF-Ag3.

#### Chronoamperometry (CA)

**Fig. 8:**
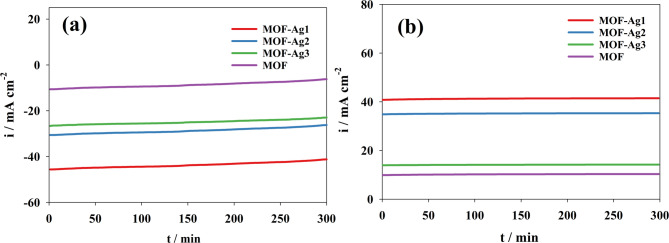
Chronoamperogram of the modified GC/MOF and MOF-Ag (MOF-Ag1, MOF-Ag2 and MOF-Ag3) for (**a**) HER and (**b**) OER in acidic medium.

Using chronoamperometry at a steady voltage, the stability of the modified MOF and MOF–Ag electrodes for gas generation was assessed in an acidic medium. Figure 8a shows the modified electrode surface could withstand hydrogen generation at a potential of − 1 V (vs. RHE) in 1 M H₂SO₄. On the other hand, oxygen evolution was measured for five hours at a potential of 2.4 V (vs. RHE) for the four electrodes, as shown in Fig. 8b. Consequently, the electrode current remained relatively stable during the 5 h test, indicating good electrochemical durability under constant applied potential conditions. Although longer chronoamperometric measurements are generally recommended for comprehensive stability evaluation, the present study was mainly designed to investigate the effect of applying a constant potential on the electrochemical behavior of the prepared electrodes.

#### Linear sweep voltammetry (LSV)

**Fig. 9:**
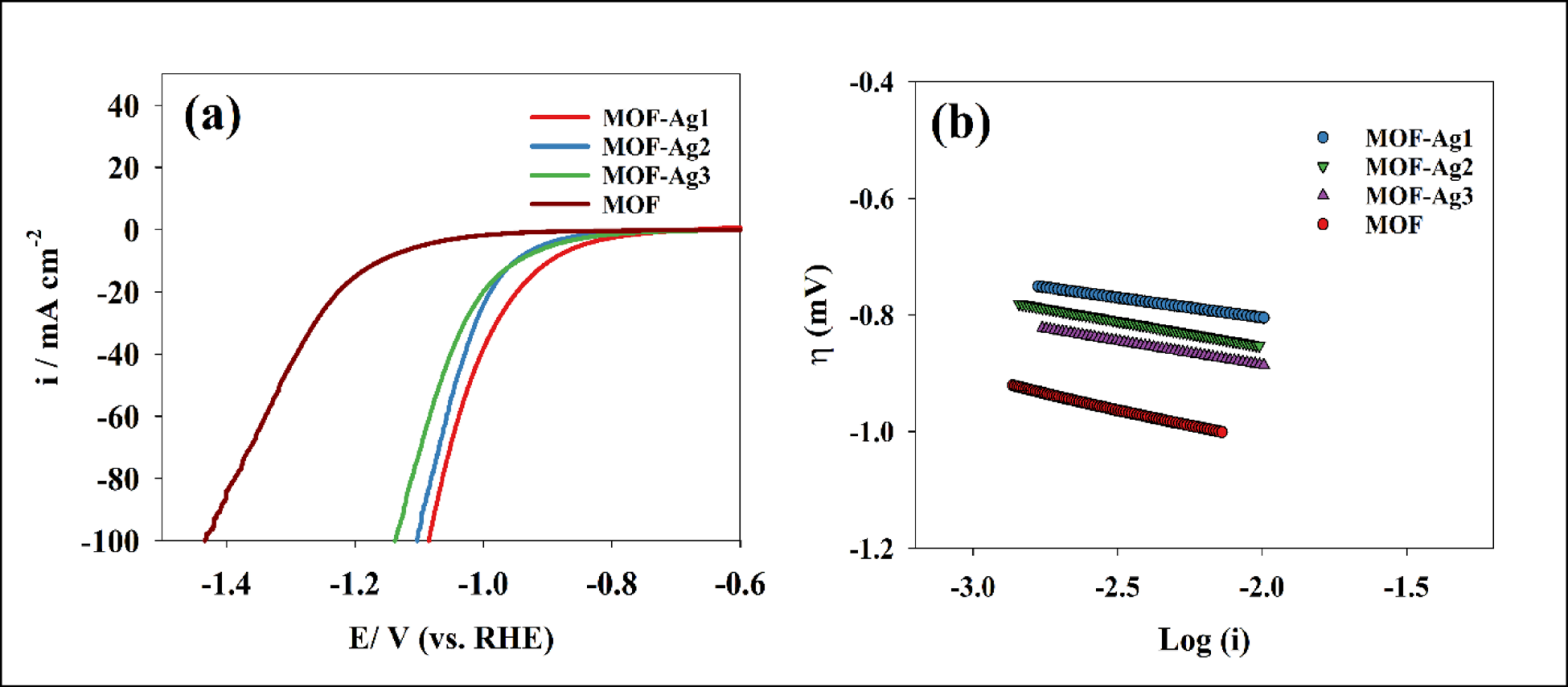
(**a**) LSV of GC/MOF and MOF-Ag (MOF-Ag1, MOF-Ag2 and MOF-Ag3) in 1M H_2_SO_4_ for HER and (**b**) Tafel Plot of HER.

The primary goal of this study was to explore the hydrogen evolution reaction (HER) mechanisms on surfaces modified by GC/MOF and MOF-Ag (MOF-Ag1, MOF-Ag2, and MOF-Ag3). This was achieved by conducting linear sweep voltammetry (LSV) experiments in a 1 M H₂SO₄ solution, as shown in Fig. 9a. The onset potential, which refers to the potential at which hydrogen evolution starts, was measured for each surface: MOF at ‒0.91 V, MOF-Ag1 at ‒0.74 V, MOF-Ag2 at ‒0.8 V, and MOF-Ag3 at ‒0.81 V (vs. RHE). These values indicate the voltage at which the electrochemical reaction begins, and lower (more negative) onset potentials generally correlate with better catalyst performance. The fact that MOF-Ag1 exhibits the most negative onset potential (‒0.74 V) suggests that it is the most efficient catalyst among the materials tested, requiring the least energy to initiate hydrogen evolution.

Thus, the HER process generally proceeds through the Volmer step followed by either the Heyrovsky step or the Tafel step:

1 $$MOF - Ag{\text{ }} + {\text{ }}H_{3} O^{ + } ~ + {\text{ }}e^{ - } ~~MOF - Ag - H{\text{ }} + {\text{ }}H_{2} O$$

2 $$MOF - Ag - H{\text{ }} + {\text{ }}H_{3} O^{ + } ~~ + {\text{ }}e^{ - } ~~~H_{2} + {\text{ }}H_{2} O{\text{ }} + {\text{ }}MOF - Ag$$

3 $$MOF - Ag - H{\text{ }} + {\text{ }}MOF - Ag - H~~H_{2} + {\text{ }}2MOF - Ag$$

The peak current density for each surface was as follows: 10 mA cm⁻² at ‒1.16 V for MOF, 10 mA cm⁻² at ‒0.88 V for MOF-Ag1, 10 mA cm⁻² at ‒0.9 V for MOF-Ag2, and 10 mA cm⁻² at ‒0.94 V for MOF-Ag3. This data reveals that MOF-Ag1 not only starts the HER process at the lowest overpotential, but also reaches the peak current at a less negative potential compared to the other surfaces, further suggesting its superior catalytic behavior. Lower overpotentials at peak currents are indicative of higher catalytic efficiency, as they imply less energy is required for the hydrogen evolution process to occur.

The exchange current densities ($\:{j}_{0}$) provide additional insight into the catalysts’ effectiveness. These values, representing the rate of reaction at equilibrium (where there is no net current), were measured as follows: $\:1.36\times\:{10}^{-12}$A cm⁻² for MOF, $\:8.1\times\:{10}^{-11}$A cm⁻² for MOF-Ag1, $\:1.17\times\:{10}^{-11}$A cm⁻² for MOF-Ag2, and $\:6.7\times\:{10}^{-12}$A cm⁻² for MOF-Ag3. The exchange current density is crucial in determining the catalytic activity; a higher $\:{j}_{0}$indicates a more active catalyst. MOF-Ag1 shows a significantly higher exchange current density compared to the others, reinforcing its superior catalytic efficiency. MOF has the lowest exchange current density, indicating a weaker catalytic performance for HER on this surface.

To better understand the kinetic behavior of the HER process, Tafel polarization curves were analyzed, as depicted in Fig. 9b. The Tafel slope provides information about the rate-limiting step in the hydrogen evolution process. A lower Tafel slope typically indicates a more efficient reaction mechanism. The Tafel slopes for each surface were calculated as 150 mV dec⁻¹ for MOF, 76 mV dec⁻¹ for MOF-Ag1, 86 mV dec⁻¹ for MOF-Ag2, and 91 mV dec⁻¹ for MOF-Ag3. MOF-Ag1 again outperforms the other surfaces, with the lowest Tafel slope, suggesting that it facilitates the HER process more efficiently, likely through a faster rate-determining step. This supports the conclusion that MOF-Ag1 is the most effective catalyst for hydrogen evolution in this study.  The comparison between our HER work and other in literature is reported in Table 2.

**Table 2:** Comparison between different surfaces for HER.

Surface	ƞ (V) @ (10 mA cm^− 2^)	Tafel Slope (mV dec^− 1^)	Electrolyte	Ref.
PS-Ag	−0.349 (vs. RHE)	188	H_2_SO_4_ (0.5 M)	^75^
PPy/Ag	−0.459(vs. RHE)	163	H_2_SO_4_ (0.5 M)	^76^
NF-8/Ag_2_Se-CoSe_2_	− 0.085(vs. RHE)	98	KOH (1.0 M)	^77^
0.5Ag/Ag_2_S	−0.37(vs. RHE)	150	H_2_SO_4_ (0.5 M)	^78^
Green-Ag nanoparticle	–0.70(vs. RHE)	61	H_2_SO_4_ (1.0 M)	^65^
MOF-Ag1	−0.88(vs. RHE)	76	H_2_SO_4_ (1.0 M)	This work

**Fig. 10:**
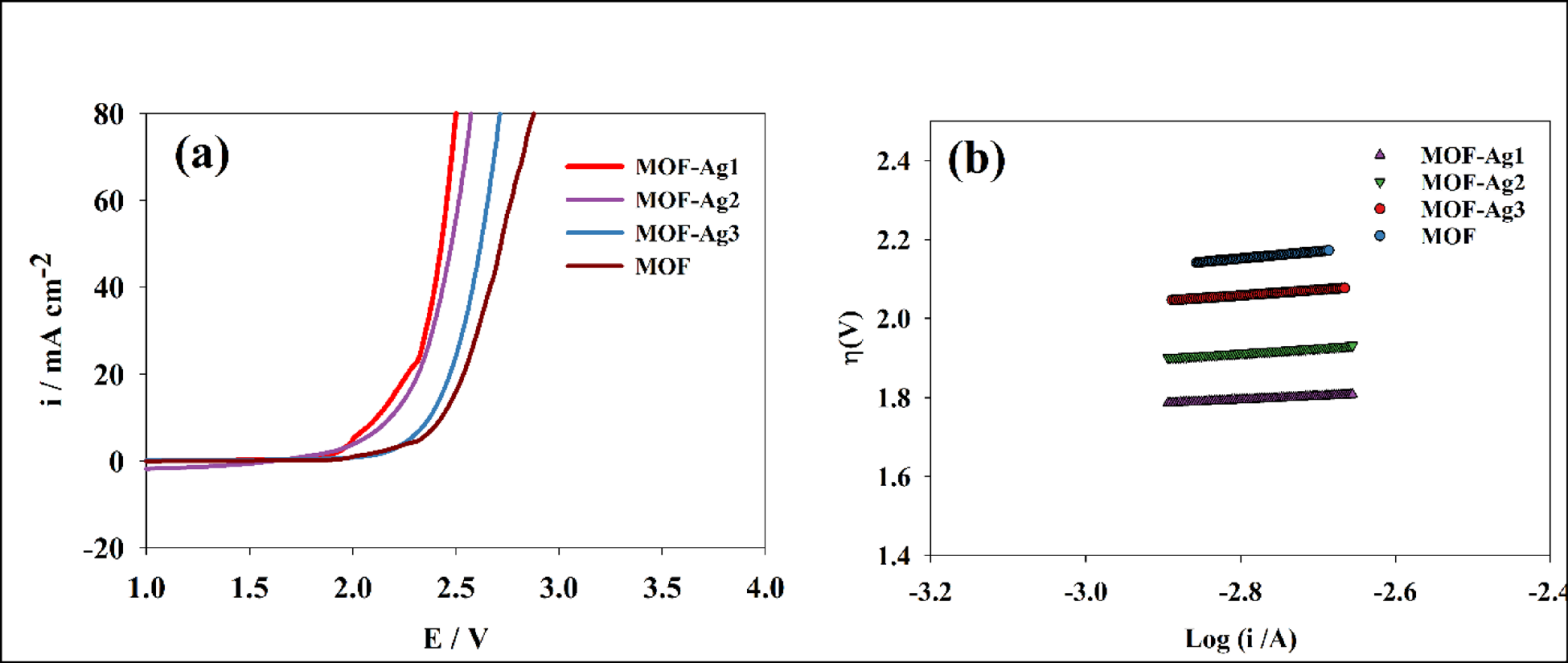
(**a**) LSV of GC/MOF and MOF-Ag (MOF-Ag1, MOF-Ag2 and MOF-Ag3) in 1 M H_2_SO_4_ for OER and (**b**) Tafel Plot of OER.

Figure 10a illustrates the oxygen evolution reaction (OER) observed on the GC/MOF and MOF-Ag (MOF-Ag1, MOF-Ag2, and MOF-Ag3) surfaces in the presence of a 1 M H₂SO₄ solution. The onset potentials, which indicate the voltage required to start the OER, were recorded at 2.29, 1.8, 1.9, and 2.1 V (vs. RHE) for GC/MOF, MOF-Ag1, MOF-Ag2, and MOF-Ag3, respectively. These values provide important information about the efficiency of the catalysts: lower onset potentials are generally indicative of better catalytic performance, as they signify that less energy is required to initiate the reaction. Among the tested surfaces, MOF-Ag1 exhibited the lowest onset potential (1.8 V), suggesting that it is the most efficient in facilitating the OER. This lower onset potential reflects the superior ability of MOF-Ag1 to activate the oxygen evolution process compared to the other materials.

In addition to the onset potential, the current peaks for the different surfaces were measured at potentials of 2.5 V for GC/MOF, 2.07 V for MOF-Ag1, 2.12 V for MOF-Ag2, and 2.18 V for MOF-Ag3, at a current density of 10 mA cm⁻². The current peak corresponds to the maximum current achieved during the reaction and is used as an indicator of the catalyst’s overall performance. MOF-Ag1 once again shows an advantage, reaching the current peak at the lowest overpotential (2.07 V), which suggests that it requires less energy to drive the reaction to a high current density compared to the other surfaces.

The generally accepted OER pathway can be described as follows:

4 $$MOF - Ag~ + {\text{ }}H_{2} O~MOF - Ag - OH_{{ads}} + {\text{ }}H^{ + } + {\text{ }}e^{ - }$$

5 $$MOF - Ag - OH_{{ads}} ~MOF - Ag - O_{{ads}} + {\text{ }}H^{ + } + {\text{ }}e^{ - }$$

6 $$MOF - Ag - OH_{{ads}} + {\text{ }}MOF - Ag - OH_{{ads}} MOF - Ag - O_{{ads}} ~ + {\text{ }}MOF - Ag{\text{ }} + {\text{ }}H_{2} O$$

7 $$MOF - Ag - O_{{ads}} ~ + {\text{ }}MOF - Ag - O_{{ads}} ~~~2MOF - Ag{\text{ }} + {\text{ }}O_{2}$$

The current densities for the different surfaces were measured and compared: $\:7.2\times\:{10}^{-13}$A cm⁻² for MOF, $\:9.1\times\:{10}^{-12}$A cm⁻² for MOF-Ag1, $\:2.32\times\:{10}^{-12}$A cm⁻² for MOF-Ag2, and $\:3.7\times\:{10}^{-12}$A cm⁻² for MOF-Ag3. These values reflect the surfaces’ catalytic activity for OER, with higher current densities indicating better performance. MOF-Ag1 exhibits the highest current density ($\:9.1\times\:{10}^{-12}$ A cm⁻²), which aligns with the lower onset potential and current peak values, further confirming its superior catalytic behavior. In contrast, MOF exhibits the lowest current density, indicating relatively poor OER performance compared to the MOF-Ag-modified surfaces.

The Tafel slopes for the OER on the modified surfaces were calculated from the Tafel plots shown in Fig. 10b. The calculated Tafel slopes were 178 mV dec⁻¹ for MOF, 90 mV dec⁻¹ for MOF-Ag1, 128 mV dec⁻¹ for MOF-Ag2, and 138 mV dec⁻¹ for MOF-Ag3. The Tafel slope is a key parameter that provides insight into the reaction mechanism and the rate-determining step of the OER. Lower Tafel slopes suggest a faster reaction rate and a more efficient catalyst. MOF-Ag1 again stands out with the lowest Tafel slope of 90 mV dec⁻¹, which is indicative of a more efficient OER process. This suggests that MOF-Ag1 facilitates the reaction more effectively, possibly through a more favorable rate-determining step. On the other hand, MOF has the highest Tafel slope (178 mV dec⁻¹), indicating that its OER process is slower and less efficient compared to the MOF-Ag-modified surfaces. Table 3 includes the comparison between our OER work and other reported in literature. 

**Table 3:** Comparison between different surfaces for OER.

Surface	ƞ (V) @ (10 mA cm^− 2^)	Tafel Slope (mV dec^− 1^)	Electrolyte	Ref.
NiFe LDH	0.35 (vs. RHE)	40	KOH (1.0 M)	^79^
CoFe LDH	0.36 (vs. RHE)	49	KOH (0.1 M)	^80^
CuCo_2_O_4_/N-rGO	0.36(vs. RHE)	64	KOH (1.0 M)	^81^
Co_3_S_4_@MoS_2_	0.33(vs. RHE)	59	KOH (1.0 M)	^82^
Ni_5_Mn-LDH- MWCNT	0.35(vs. RHE)	83.5	KOH (1.0 M)	^83^
Green-Ag nanoparticle	0.3 (vs. RHE)	68	H_2_SO_4_ (1.0 M)	^65^
MOF-Ag1	0.84 (vs. RHE)	90	H_2_SO_4_ (1.0 M)	This work

#### Electrochemical impedance spectroscopy (EIS)

**Fig. 11:**
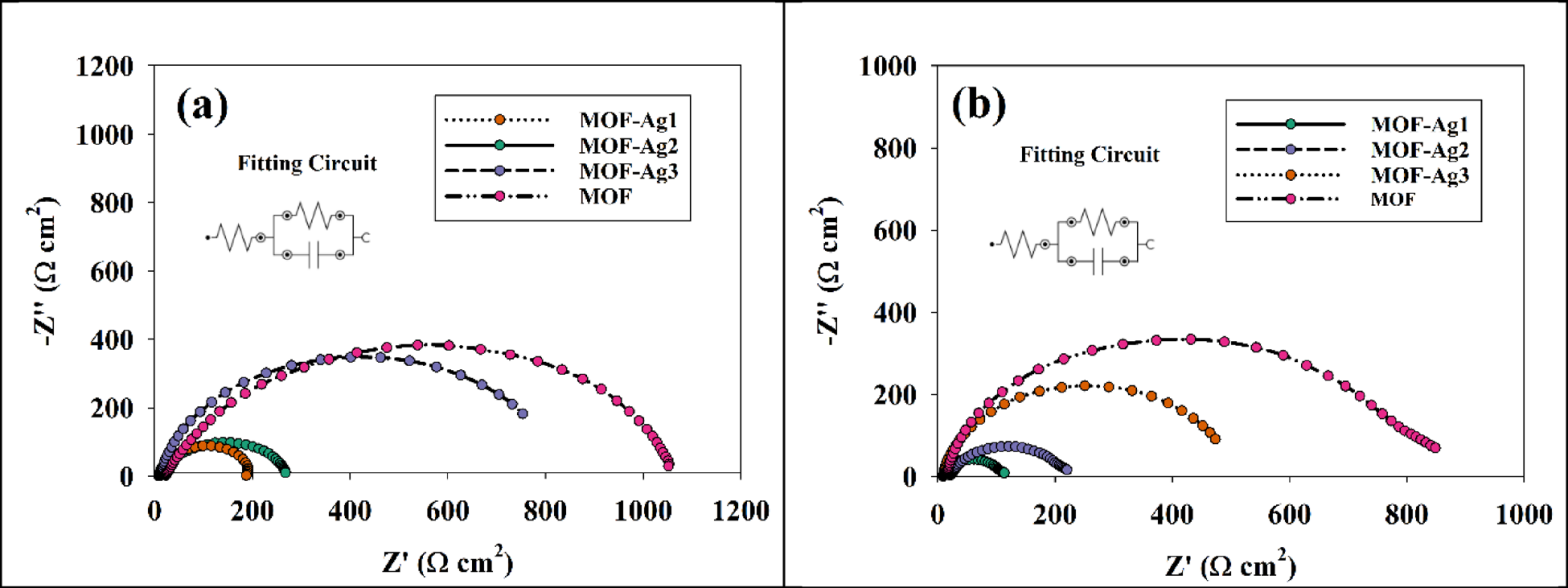
Nyquist plot of GC/MOF and MOF-Ag (MOF-Ag1, MOF-Ag2 and MOF-Ag3) for (**a**) HER, and (**b**) OER.

The hydrogen and oxygen evolution reactions at the modified GC/MOF and MOF–Ag (MOF–Ag1, MOF–Ag2, and MOF–Ag3) electrodes were investigated using EIS. As demonstrated in Fig. 9A, HER measurements were performed in 1 M H₂SO₄ at a fixed AC potential of − 0.8 V (vs. RHE) for the MOF electrode and − 1.0 V (vs. RHE) for the MOF–Ag electrodes, providing a summary of the fitting parameters in Table 4. The corresponding Nyquist plots displayed semi-circular features, indicating charge-transfer behavior. Similarly, Fig. 11b presents the Nyquist response for the same electrodes under OER conditions, measured at 2.1 V (vs. RHE). The resulting semi-circles in the Nyquist diagrams reflect the charge-transfer mechanism, and the associated fitting parameters are provided in Table 5. The goodness factors were 0.013–0.068 for HER and 0.009–0.016 for OER. The fitting circuit was added to the inset in Fig. 11.

**Table 4:** Fitting parameters for HER.

Electrode	Rs (Ω)	Rc (Ω)	C(F)
MOF-Ag1	9.5	177	0.00000651
MOF-Ag2	10.8	267	0.00000387
MOF-Ag3	11.3	755	0.00000369
MOF	12.4	1060	0.00000235

**Table 5:** Fitting parameters for OER.

Electrode	Rs (Ω)	Rc (Ω)	C(F)
MOF-Ag1	9.01	108	0.00000706
MOF-Ag2	21.6	223	0.00000631
MOF-Ag3	9.48	473	0.00000432
MOF	10.6	815	0.00000398

## Conclusion

This study confirms that the incorporation of Ag nanoparticles into a cerium-based MOF, synthesized via a green route, effectively enhances the intrinsic limitations of MOFs, particularly their low electrical conductivity and limited active site accessibility. The MOF–Ag composites exhibit improved electrocatalytic performance toward both HER and OER, as evidenced by lower overpotentials at 10 mA cm⁻² and reduced Tafel slopes compared to the pristine MOF, indicating accelerated charge transfer kinetics and more favorable reaction pathways. The observed enhancement can be attributed to the synergistic interaction between the Ce-MOF framework and Ag nanoparticles, which facilitates electron transport and increases the density of electrochemically active sites. Furthermore, BET and contact angle analyses confirm that the mesoporous structure and hydrophilic surface promote efficient electrolyte diffusion and interfacial charge transfer, contributing to the overall catalytic efficiency. The stability of the electrodes over 5 h of continuous operation further demonstrates the structural robustness of the composites under electrochemical conditions. These findings establish a direct correlation between physicochemical properties and electrocatalytic behavior, providing a rational basis for the design of MOF-based hybrid catalysts with optimized performance for water-splitting applications.

## Supplementary Information

Below is the link to the electronic supplementary material.

Supplementary Material 1

## Data Availability

All data generated or analyzed during this study are included in this published article [and its supplementary information files].

## References

[1] Abazari, R., Goscianska, J., Naderi, M., Liu, M. & Sanati, S. Trifunctional noble-metal-free multi-site electrocatalysts based on NiMn-LDH/CuCo2S4/rGO for energy-saving hydrogen generation via UOR/HER/OER. *Journal Mater. Chem. A* (2026).

[2] Sanati, S., Cordes, D. B., Slawin, A. M., Qian, J. & Abazari, R. Highly conductive non-calcined 2D Cu0. 3Co0. 7 bimetallic–organic framework for urea electrolysis in simulated seawater. *Inorg. Chem.* **64** (1), 510–518 (2024). 39705333 10.1021/acs.inorgchem.4c05162

[3] Sanati, S. et al. Energy-Saving Hydrogen Production from Electrocatalytic Oxidation of Urea in Seawater over Mixed-Linker Mn1–x Co x Metal–Organic Frameworks with Open Metal Sites. *Inorg. Chem.* **64** (40), 20507–20516 (2025). 41032572 10.1021/acs.inorgchem.5c03912

[4] Zhang, W. et al. Dendritic Fe-based polyoxometalates@ metal–organic framework (MOFs) combined with ZnO as a novel photoanode in solar cells. *J. Mater. Sci.: Mater. Electron.* **29** (2), 1623–1629 (2018).

[5] Bhanja, P. et al. A new porous polymer for highly efficient capacitive energy storage. *ACS Sustain. Chem. Eng.* **6** (1), 202–209 (2018).

[6] Kim, J. H. et al. High efficiency and stable solid-state fiber dye-sensitized solar cells obtained using TiO2 photoanodes enhanced with metal organic frameworks. *J. Energy Chem.* **67**, 458–466 (2022).

[7] Lin, K. S., Adhikari, A. K., Ku, C. N., Chiang, C. L. & Kuo, H. Synthesis and characterization of porous HKUST-1 metal organic frameworks for hydrogen storage. *Int. J. Hydrog. Energy*. **37** (18), 13865–13871 (2012).

[8] Yang, H. et al. Metal–organic framework coated titanium dioxide nanorod array p–n heterojunction photoanode for solar water-splitting. *Nano Res.* **12** (3), 643–650 (2019).

[9] Ban, Y. et al. Direct production of hydrogen-enriched syngas by calcium-catalyzed steam gasification of Shengli lignite/chars: Structural evolution. *Int. J. Hydrog. Energy*. **45** (15), 8357–8368 (2020).

[10] Hefnawy, M. A., Fadlallah, S. A., El-Sherif, R. M. & Medany, S. S. Synergistic effect of Cu-doped NiO for enhancing urea electrooxidation: Comparative electrochemical and DFT studies. *J. Alloys Compd.* **896**, 162857 (2022).

[11] Lakshmi, K. S., Vedhanarayanan, B. & Lin, T. W. Electrocatalytic hydrogen and oxygen evolution reactions: Role of two-dimensional layered materials and their composites. *Electrochim. Acta* **447**, 142119 (2023).

[12] Sowmya, S. et al. Synthesis, crystal structure and electrocatalytic hydrogen evolution reaction studies of cobaloximes with diphenylglyoxime and carboxylic acid functionalized neutral bases. *Polyhedron* **238**, 116394 (2023).

[13] Hefnawy, M. A., Fadlallah, S. A., El-Sherif, R. M. & Medany, S. S. Systematic DFT studies of CO-tolerance and CO oxidation on Cu-doped Ni surfaces. *J. Mol. Graph. Model.* **118**, 108343 (2023). 36208590 10.1016/j.jmgm.2022.108343

[14] Alamro, F. S. et al. Chitosan supports boosting NiCo2O4 for catalyzed urea electrochemical removal application. *Polymers* **15** (14), 3058 (2023). 37514447 10.3390/polym15143058 PMC10384518

[15] Li, L., Wang, P., Shao, Q. & Huang, X. Metallic nanostructures with low dimensionality for electrochemical water splitting. *Chem. Soc. Rev.* **49** (10), 3072–3106 (2020). 32309830 10.1039/d0cs00013b

[16] Amin, N. U. et al. Electrocatalytic performance of hetrostructured MoS2/Ag2S/Ag nanocomposites for hydrogen evolution reaction. *Mater. Sci. Semicond. Process.* **162**, 107519 (2023).

[17] Hayashi, Y., Ebato, Y., Onishi, R. & Takizawa, H. Sonochemical decomposition of noble metal oxides and sonochemical alloying of gold–silver systems. *Ultrason. Sonochem.* **89**, 106115 (2022). 35988292 10.1016/j.ultsonch.2022.106115 PMC9418546

[18] Beshkar, F., Zinatloo-Ajabshir, S. & Salavati-Niasari, M. Preparation and characterization of the CuCr2O4 nanostructures via a new simple route. *J. Mater. Sci. Mater. Electron.* **26**(7), 5043–5051 (2015).

[19] Zinatloo-Ajabshir, S. & Salavati-Niasari, M. Novel poly (ethyleneglycol)-assisted synthesis of praseodymium oxide nanostructures via a facile precipitation route. *Ceram. Int.* **41** (1), 567–575 (2015).

[20] Tan, X. et al. Application of MOF-derived transition metal oxides and composites as anodes for lithium-ion batteries. *Inorg. Chem. Front.* **7** (24), 4939–4955 (2020).

[21] Wu, R. et al. MOF-templated formation of porous CuO hollow octahedra for lithium-ion battery anode materials. *J. Mater. Chem. A*. **1** (37), 11126–11129 (2013).

[22] Jayaramulu, K. et al. Shape‐assisted 2D MOF/graphene derived hybrids as exceptional lithium‐ion battery electrodes. *Adv. Funct. Mater.* **29**(38), 1902539 (2019).

[23] Sheberla, D. et al. Conductive MOF electrodes for stable supercapacitors with high areal capacitance. *Nat. Mater.* **16**(2), 220–224 (2017). 27723738 10.1038/nmat4766

[24] Xia, H. et al. 2D MOF nanoflake-assembled spherical microstructures for enhanced supercapacitor and electrocatalysis performances. *Nano-micro Lett.* **9** (4), 43 (2017). 10.1007/s40820-017-0144-6 PMC6199045 30393738

[25] Liu, B., Shioyama, H., Jiang, H., Zhang, X. & Xu, Q. Metal–organic framework (MOF) as a template for syntheses of nanoporous carbons as electrode materials for supercapacitor. *Carbon* **48** (2), 456–463 (2010).

[26] Jin, Y. et al. Surface functionalization of carbon cloth with conductive Ni/Fe-MOFs for highly efficient oxygen evolution. *Surf. Interfaces* **33**, 102294 (2022).

[27] Zhang, M., Dai, Q., Zheng, H., Chen, M. & Dai, L. Novel MOF-derived Co@ N‐C bifunctional catalysts for highly efficient Zn–air batteries and water splitting. *Adv. Mater.* **30**(10), 1705431 (2018). 10.1002/adma.201705431 29349841

[28] Mohanty, A. et al. An extensive review on three-dimension architectural metal-organic frameworks towards supercapacitor application. *J. Power Sources* **488**, 229444 (2021).

[29] Wagner, M., Lin, K. Y. A., Oh, W. D. & Lisak, G. Metal-organic frameworks for pesticidal persistent organic pollutants detection and adsorption–a mini review. *J. Hazard. Mater.* **413**, 125325 (2021). 33601143 10.1016/j.jhazmat.2021.125325

[30] Heo, D. Y., Do, H. H., Ahn, S. H. & Kim, S. Y. Metal-organic framework materials for perovskite solar cells. *Polymers* **12**(9), 2061 (2020). 32927727 10.3390/polym12092061 PMC7569814

[31] Hwang, Y. K. et al. Amine grafting on coordinatively unsaturated metal centers of MOFs: Consequences for catalysis and metal encapsulation. *Angew. Chem.* **120**(22), 4212–4216 (2008). 10.1002/anie.200705998 18435442

[32] Eddaoudi, M. et al. Systematic design of pore size and functionality in isoreticular MOFs and their application in methane storage. *Science* **295** (5554), 469–472 (2002). 11799235 10.1126/science.1067208

[33] Thornton, A. W., Nairn, K. M., Hill, J. M., Hill, A. J. & Hill, M. R. Metal– organic frameworks impregnated with magnesium-decorated fullerenes for methane and hydrogen storage. *J. Am. Chem. Soc.* **131** (30), 10662–10669 (2009). 19583258 10.1021/ja9036302

[34] Kim, M., Cahill, J. F., Fei, H., Prather, K. A. & Cohen, S. M. Postsynthetic ligand and cation exchange in robust metal–organic frameworks. *J. Am. Chem. Soc.* **134** (43), 18082–18088 (2012). 23039827 10.1021/ja3079219

[35] Yang, S. J. et al. Preparation and enhanced hydrostability and hydrogen storage capacity of CNT@ MOF-5 hybrid composite. *Chem. Mater.* **21** (9), 1893–1897 (2009).

[36] Petit, C. & Bandosz, T. J. MOF–graphite oxide composites: combining the uniqueness of graphene layers and metal–organic frameworks. *Adv. Mater.* **21** (46), 4753–4757 (2009).

[37] Ahmed, I. & Jhung, S. H. Composites of metal–organic frameworks: preparation and application in adsorption. *Mater. Today*. **17** (3), 136–146 (2014).

[38] Juan-Alcañiz, J., Gascon, J. & Kapteijn, F. Metal–organic frameworks as scaffolds for the encapsulation of active species: state of the art and future perspectives. *J. Mater. Chem.* **22** (20), 10102–10118 (2012).

[39] Bradshaw, D., Garai, A. & Huo, J. Metal–organic framework growth at functional interfaces: thin films and composites for diverse applications. *Chem. Soc. Rev.* **41** (6), 2344–2381 (2012). 22182916 10.1039/c1cs15276a

[40] Work, W. J., Horie, K., Hess, M. & Stepto, R. F. T. Definition of terms related to polymer blends, composites, and multiphase polymeric materials (IUPAC Recommendations 2004). *Pure Appl. Chem.* **76**(11), 1985–2007 (2004).

[41] Wee, L. H. et al. Convenient synthesis of Cu3 (BTC) 2 encapsulated Keggin heteropolyacid nanomaterial for application in catalysis. *Chem. Commun.* **46** (43), 8186–8188 (2010). 10.1039/c0cc01447h 20927469

[42] Jahan, M., Bao, Q., Yang, J. X. & Loh, K. P. Structure-directing role of graphene in the synthesis of metal– organic framework nanowire. *J. Am. Chem. Soc.* **132** (41), 14487–14495 (2010). 20863117 10.1021/ja105089w

[43] Petit, C. & Bandosz, T. J. Enhanced adsorption of ammonia on metal-organic framework/graphite oxide composites: analysis of surface interactions. *Adv. Funct. Mater.* **20** (1), 111–118 (2010).

[44] Petit, C. et al. Toward understanding reactive adsorption of ammonia on Cu-MOF/graphite oxide nanocomposites. *Langmuir* **27** (21), 13043–13051 (2011). 21970728 10.1021/la202924y

[45] Mustafa, D., Breynaert, E., Bajpe, S. R., Martens, J. A. & Kirschhock, C. E. Stability improvement of Cu 3 (BTC) 2 metal–organic frameworks under steaming conditions by encapsulation of a Keggin polyoxometalate. *Chem. Commun.* **47** (28), 8037–8039 (2011). 10.1039/c1cc12341f 21674103

[46] O’Neill, L. D., Zhang, H. & Bradshaw, D. Macro-/microporous MOF composite beads. *J. Mater. Chem.* **20**(27), 5720–5726 (2010).

[47] Liang, D. D., Liu, S. X., Ma, F. J., Wei, F. & Chen, Y. G. A crystalline catalyst based on a porous metal-organic framework and 12‐tungstosilicic acid: Particle size control by hydrothermal synthesis for the formation of dimethyl ether. *Adv. Synth. Catal.* **353** (5), 733–742 (2011).

[48] Zinatloo-Ajabshir, S. & Salavati-Niasari, M. Preparation of magnetically retrievable CoFe2O4@ SiO2@ Dy2Ce2O7 nanocomposites as novel photocatalyst for highly efficient degradation of organic contaminants. *Compos. Part B Eng.* **174**, 106930 (2019).

[49] Zinatloo-Ajabshir, S., Morassaei, M. S., Amiri, O. & Salavati-Niasari, M. Green synthesis of dysprosium stannate nanoparticles using Ficus carica extract as photocatalyst for the degradation of organic pollutants under visible irradiation. *Ceram. Int.* **46** (5), 6095–6107 (2020).

[50] Islamoglu, T., Atilgan, A., Moon, S. Y., Peterson, G. W., DeCoste, J. B., Hall, M.,… Farha, O. K. (2017). Cerium (IV) vs zirconium (IV) based metal–organic frameworks for detoxification of a nerve agent. *Chemistry of Materials*, *29*(7), 2672–2675.

[51] Lammert, M. et al. Cerium-based metal organic frameworks with UiO-66 architecture: Synthesis, properties and redox catalytic activity. *Chem. Commun.* **51**(63), 12578–12581 (2015). 10.1039/c5cc02606g 26154160

[52] Li, K., Yang, J. & Gu, J. Salting-in species induced self-assembly of stable MOFs. *Chem. Sci.* **10** (22), 5743–5748 (2019). 31293760 10.1039/c9sc01447k PMC6568048

[53] Li, K., Yang, J., Huang, R., Lin, S. & Gu, J. Ordered large-pore MesoMOFs based on synergistic effects of triblock polymer and hofmeister ion. *Angew. Chem.* **132** (33), 14228–14232 (2020). 10.1002/anie.202006124 32400955

[54] Guo, Y. et al. Ce–UiO-66 derived CeO2 octahedron catalysts for efficient ketonization of propionic acid. *Ind. Eng. Chem. Res.* **59**(39), 17269–17278 (2020).

[55] Zinatloo-Ajabshir, S., Emsaki, M. & Hosseinzadeh, G. Innovative construction of a novel lanthanide cerate nanostructured photocatalyst for efficient treatment of contaminated water under sunlight. *J. Colloid Interface Sci.* **619**, 1–13 (2022). 35367923 10.1016/j.jcis.2022.03.112

[56] Al-Gethami, W., Alhashmialameer, D., Al-Qasmi, N., Ismail, S. H. & Sadek, A. H. Design of a novel nanosensors based on green synthesized CoFe2O4/Ca-alginate nanocomposite-coated QCM for rapid detection of Pb (II) ions. *Nanomaterials* **12** (20), 3620 (2022). 36296809 10.3390/nano12203620 PMC9610289

[57] Widatalla, H. A. et al. Green synthesis of silver nanoparticles using green tea leaf extract, characterization and evaluation of antimicrobial activity. *Nanoscale Adv.* **4** (3), 911–915 (2022). 36131825 10.1039/d1na00509j PMC9419201

[58] Sökmen, M., Alomar, S. Y., Albay, C. & Serdar, G. Microwave assisted production of silver nanoparticles using green tea extracts. *J. Alloys Compd.* **725**, 190–198 (2017).

[59] Yassin, J. M., Taddesse, A. M. & Sánchez-Sánchez, M. Room temperature synthesis of high-quality Ce (IV)-based MOFs in water. *Microporous Mesoporous Mater.* **324**, 111303 (2021).

[60] Meng, Y. A sustainable approach to fabricating Ag nanoparticles/PVA hybrid nanofiber and its catalytic activity. *Nanomaterials* **5** (2), 1124–1135 (2015). 28347055 10.3390/nano5021124 PMC5312901

[61] Hefnawy, M. A., Nafady, A., Mohamed, S. K. & Medany, S. S. Facile green synthesis of Ag/carbon nanotubes composite for efficient water splitting applications. *Synth. Met.* **294**, 117310 (2023).

[62] Jacobsen, J., Ienco, A., D’Amato, R., Costantino, F. & Stock, N. The chemistry of Ce-based metal–organic frameworks. *Dalton Trans.* **49** (46), 16551–16586 (2020). 33146175 10.1039/d0dt02813d

[63] Zhang, H., Liu, Y. & Wang, J. Silver nanoparticle-embedded metal–organic frameworks with enhanced crystallinity and stability. *CrystEngComm* **22** (45), 7801–7810 (2020).

[64] Ahmed Malik, W. M. et al. A facile synthesis of CeO2 from the GO@ Ce-MOF precursor and its efficient performance in the oxygen evolution reaction. *Front. Chem.* **10**, 996560 (2022). 36277339 10.3389/fchem.2022.996560 PMC9585184

[65] Ibrahim, N. M. et al. Chemical and green synthesis of silver nanoparticles and their use as an electrocatalyst for water splitting. *Int. J. Hydrog. Energy*. **159**, 150536 (2025).

[66] Li, X., Zhang, Y., Chen, Y. & Zhao, J. Photo-induced preparation of Ag@MOF-801 composite and its catalytic performance. *Catalysts* **12** (5), 533 (2022).

[67] Nikmehr, S., Kazemzad, M., Sabzehmeidani, M. M., Nikzad, L. & Ebadzadeh, T. Structural characteristics of Zn-MOFs and derived zinc oxide by X-ray diffraction peak analysis fabricated by mechanical and hydrothermal methods. *OpenNano* **16**, 100203 (2024).

[68] Elshafei, M. F., Mostafa, M. R., Khalf-Alla, P. A., Mohamed, G. G. & Fouad, O. A. Kinetic and isotherm study of Ni-MOF/Magnetite nanoparticles adsorption capacity as green synthesized adsorbent towards rhodochrome (Kammererite). *Sci. Rep.* **15** (1), 44669 (2025). 41457194 10.1038/s41598-025-29707-7 PMC12749104

[69] Nasser, N., Wahsh, M. M., Rizk, M. S., Mohamed, G. G. & Fouad, O. A. Effect of plant waste materials as pore-forming agents on the preparation and characterization of macroporous cordierite–mullite–zirconia ceramic composites. *BMC chemistry*. (2025). 10.1186/s13065-025-01696-8 PMC12829238 41420193

[70] Georgy, A. N., Omar, M. A., Mostafa, M. R., Mohamed, G. G. & Fouad, O. A. Removal of 2, 4 di-nitrophenol by using modified spinel aluminate/chitosan nanoparticles composites. *Sci. Rep.*10.1038/s41598-025-28057-8 (2025). 41339397 10.1038/s41598-025-28057-8 PMC12678815

[71] Georgy, A. N., Omar, M. A., Mostafa, M. R., Mohamed, G. G. & Fouad, O. A. Enhanced adsorption of organic pollutants from wastewater using Chitosan and MAS/CS nanoparticles (MAS/CS-NPs). *Journal Mol. Structure*, 145793. (2026).

[72] Tang, C., Sun, A., Xu, Y., Wu, Z. & Wang, D. High specific surface area Mo2C nanoparticles as an efficient electrocatalyst for hydrogen evolution. *J. Power Sources*. **296**, 18–22 (2015).

[73] Gujral, H. S. et al. Nanoporous TiCN with High Specific Surface Area for Enhanced Hydrogen Evolution Reaction. *ACS Appl. Nano Mater.* **5** (9), 12077–12086 (2022).

[74] Zayed, M. A., Abbas, A. A., Mahmoud, W. H., Ali, A. E. & Mohamed, G. G. Development and surface characterization of a bis (aminotriazoles) derivative based renewable carbon paste electrode for selective potentiometric determination of Cr (III) ion in real water samples. *Microchem. J.* **159**, 105478 (2020).

[75] Bai, Y. Y. et al. A silver catalyst with a high-energy surface prepared by plasma spraying for the hydrogen evolution reaction. *Chem. Commun.* **58** (17), 2878–2881 (2022). 10.1039/d1cc06892j 35132980

[76] Sabalová, M. et al. Electrocatalytic hydrogen evolution in acidic media using electrodeposited Ag/PPy and Ni/PPy hybrid materials. *Chem. Pap.* **71** (2), 513–523 (2017).

[77] Yang, L., Zhao, Y., Zhu, L. & Xia, D. Superionic conductor Ag2Se modulated CoSe2 nanosheets prepared via monometallic cation release for efficient pH-universal water electrolysis into hydrogen. *J. Colloid Interface Sci.* **627**, 503–515 (2022). 35870403 10.1016/j.jcis.2022.07.076

[78] Cova, C. M. et al. Microwave-assisted preparation of Ag/Ag_2_S carbon hybrid structures from pig bristles as efficient HER catalysts. *J. Mater. Chem. A.* **6**(43), 21516–21523 (2018).

[79] Song, F. & Hu, X. Exfoliation of layered double hydroxides for enhanced oxygen evolution catalysis. *Nat. Commun.* **5**(1), 4477 (2014). 25030209 10.1038/ncomms5477

[80] Yang, F. et al. Synergistic effect of cobalt and iron in layered double hydroxide catalysts for the oxygen evolution reaction. *ChemSusChem* **10** (1), 156–165 (2017). 27865059 10.1002/cssc.201601272

[81] Bikkarolla, S. K. & Papakonstantinou, P. CuCo2O4 nanoparticles on nitrogenated graphene as highly efficient oxygen evolution catalyst. *J. Power Sources*. **281**, 243–251 (2015).

[82] Guo, Y., Tang, J., Qian, H., Wang, Z. & Yamauchi, Y. One-pot synthesis of zeolitic imidazolate framework 67-derived hollow Co3S4@ MoS2 heterostructures as efficient bifunctional catalysts. *Chem. Mater.* **29** (13), 5566–5573 (2017).

[83] Jia, G. et al. Formation of hierarchical structure composed of (Co/Ni) Mn-LDH nanosheets on MWCNT backbones for efficient electrocatalytic water oxidation. *ACS Appl. Mater. Interfaces*. **8** (23), 14527–14534 (2016). 27214293 10.1021/acsami.6b02733

