# Supplementary material for: Synthesis of Ce-MOF/Ag composites with improved electrocatalytic activity and stability for sustainable water splitting
Source: Sci Rep. 2026 Jul 12;16:21649. doi: 10.1038/s41598-026-56365-0 (PMC13357744; doi:10.1038/s41598-026-56365-0)
Supplement: Supplementary file 1 — Supplementary Material 1 [file 41598_2026_56365_MOESM1_ESM.docx]

**Synthesis of Ce-MOF/Ag Composites with Improved Electrocatalytic Activity and Stability for Sustainable Water Splitting**

Nourhan M Ibrahim^1*^, Ayman S. Eliwa^1^, Mahmoud A. Hefnawy^1^, Gehad G. Mohamed^1,2^, Wafaa M. Hosny^1^, Omar A. Fouad^1*^

1. Chemistry Department, Faculty of Science, Cairo University, 12613, Giza, Egypt.
2. Nanoscience Department, Basic and Applied Sciences Institute, Egypt-Japan University of Science and Technology, New Borg El Arab, Alexandria, 21934, Egypt

**Materials**

Silver nitrate (AgNO_3_) has CAS (7761-88-8) and purity 99%, cerium(IV) ammonium nitrate ((NH_4_)_2_Ce(NO_3_)_6_) has CAS (16774-21-3) and purity 99.9%, terephthalic acid (H_2_BDC) has CAS (100-21-0) and purity 98%, ethanol has CAS (64-17-5) and purity 99.5%, acetone has CAS (67-64-1) and purity 99%, DMF has CAS (68-12-2) and purity 99% and sulfuric acid has CAS (7664-93-9) and purity 97% were purchased from Sigma-Aldrich Chemie GmbH Eschenstrasse 5. Clove was bought from a local shop. Deionized water from all glass equipment has been saved and used in the whole preparation processes.

**Instruments**

The Bruker D8 Discover (Bruker AXS Inc., 35 KV, 30 mA) X-ray diffractometer registered the X-ray diffraction (XRD) at the Egypt Nanotechnology Center (EGNC) with a step size of 0.02 and a speed scan of 0.016. The Cu Kα radiation (λ = 1.5406 Å) was used for two hours, with 2θ varying from 5 to 50. Gas adsorption experiments have been carried out at 77 K with N_2_ serving as the adsorptive gas in order to determine BET surface area and the pore size distribution. The materials were placed under a high vacuum and evacuated for 4–12 hours before the adsorption experiments. The computation was based on the Brunauer-Emmett-Teller (BET) theory, and the analysis was carried out using a Nova Touch LX_2_ analyzer. Utilizing a SEM Model Quanta 250 FEG (Field Emission Gun) with an accelerating voltage of 30 K.V., magnification of 14x up to 1000000, and resolution for Gun.1n, the Egyptian Mineral Resources Authority Central Laboratories Sector was involved. Before testing, samples received a thin layer of gold coating. The shape and size of the generated nanoparticles were examined using transmission electron microscopy (TEM; JEOL JEM-2100, Tokyo, Japan). FT-IR Spectra. Using a PerkinElmer 1650 spectrom eter (4000−400 cm−1), FT-IR spectra have been recorded with KBr pellets. The contact angle was measured using Attention-Theta of Biolin Scientific in the Egypt Nanotechnology Center at Cairo University in El-Sheikh Zayed, Egypt, on 6th October. Electrochemical measurement has been carried out using CHl 660E electrochemical workstation, the Ag/AgCl reference electrode and glassy carbon working electrode.
